# Sailfish generate foraging opportunities for seabirds in multi-species predator aggregations

**DOI:** 10.1098/rsbl.2024.0177

**Published:** 2024-07-10

**Authors:** M. Licht, A. L. Burns, K. Pacher, S. Krause, P. Bartashevich, P. Romanczuk, M. J. Hansen, A. Y. Then, J. Krause

**Affiliations:** ^1^ Faculty of Life Sciences, Albrecht Daniel Thaer-Institute of Agricultural and Horticultural Sciences, Humboldt-Universität zu Berlin, Invalidenstrasse 42, Berlin 10115, Germany; ^2^ Cluster of Excellence ‘Science of Intelligence’, Berlin, Germany, Marchstr. 23, Berlin 10587, Germany; ^3^ Department Fish Biology, Fisheries and Aquaculture, Leibniz Institute of Freshwater Ecology and Inland Fisheries, Müggelseedamm 310, Berlin 12587, Germany; ^4^ Department of Electrical Engineering and Computer Science, Lübeck University of Applied Sciences, Lübeck 23562, Germany; ^5^ Institute for Theoretical Biology, Department of Biology, Humboldt-Universität zu Berlin, Berlin, Germany; ^6^ Institute of Biological Sciences, Faculty of Science, University of Malaya, Lembah Pantai, Kuala Lumpur 50603, Malaysia

**Keywords:** sailfish, foraging, predator interaction, schooling behaviour, seabirds

## Abstract

While various marine predators form associations, the most commonly studied are those between subsurface predators and seabirds, with gulls, shearwaters or terns frequently co-occurring with dolphins, billfish or tuna. However, the mechanisms underlying these associations remain poorly understood. Three hypotheses have been proposed to explain the prevalence of these associations: (1) subsurface predators herd prey to the surface and make prey accessible to birds, (2) subsurface predators damage prey close to the surface and thereby provide food scraps to birds, and (3) attacks of underwater predators lower the cohesion of prey groups and thereby their collective defences making the prey easier to be captured by birds. Using drone footage, we investigated the interaction between Indo-Pacific sailfish (*Istiophorus platypterus*) and terns (*Onychoprion* sp*.*) preying on schooling fish off the eastern coast of the Malaysian peninsula. Through spatio-temporal analysis of the hunting behaviour of the two predatory species and direct measures of prey cohesion we showed that terns attacked when school cohesion was low, and that this decrease in cohesion was frequently caused by sailfish attacks. Therefore, we propose that sailfish created a by-product benefit for the bird species, lending support to the hypothesis that lowering cohesion can facilitate associations between subsurface predators and seabirds.

## 1. Introduction

The open ocean harbours the largest predator aggregations on the planet [1,2]. Thousands of predators of different species can gather in the same location and simultaneously predate upon large schools of prey [2]. Despite the ecological relevance of these multi-species predator aggregations and extensive literature documenting the co-occurrence of particular combinations of predator species, their synergistic or antagonistic nature is rarely assessed (but see [3,4]). This is likely due to difficulties quantifying predator behaviours such as attack and capture rates as well as the spatio-temporal positions of predators and prey in the wild. This means that the mechanisms that underlie the antagonistic or synergistic nature of predator interactions are also poorly understood [4].

Various marine predators are known to associate with each other, including for example: dolphins and tuna [5–7], seals and billfishes [4], humans and dolphins [8], sharks and trevally [9,10], dolphins and baleen whales [11,12]. However, most commonly studied are associations of large subsurface predators (e.g. delphinids, tuna, pinnipeds) with seabirds (e.g. terns, gulls, shearwaters) [3,5,13–19]. The prevalence of these particular associations is usually explained by three proposed benefits that subsurface predators provide for seabirds: (1) subsurface predators make fish schools accessible to birds by driving them to the surface; (2) injuries inflicted on prey fish by subsurface predators make tissue and scales available to bird species that do not prey on the whole fish; (3) predator attacks on the prey school reduce its cohesion and thus reduce the collective defensive mechanisms of the fish. The first two hypotheses have been supported by direct evidence [16,20–22], however, a direct measure of prey cohesion in the wild in the context of multi-species aggregations (and thus support for the third hypothesis) is currently lacking.

The benefit that has received the most attention in the existing literature is that subsurface predators take part in collective herding; driving prey animals from the lower layers in the water column to the surface where they become accessible to birds [5,13,15,19,22–26]. Driving schooling prey to the surface is a common predator strategy in the ocean and likely benefits subsurface predators by restricting the movement and potential escape routes of prey schools. There are numerous anecdotal accounts of this behaviour [6,24,27], however, due to logistical difficulties observing predator–prey interactions at depth, quantification of predators driving prey schools to the surface has only been achieved via hydro-acoustics and animal-borne underwater cameras [20–22]. When orcas collectively herded schools of herring (*Clupea harengus*) to the surface the fish were subsequently observed to be preyed upon by common gulls (*Larus canus*) and white-tailed eagle (*Haliaeetus albicilla*) [20]. In the absence of such direct evidence, an alternative scenario could be that both predator types, avian and subsurface, encounter prey that is already at the surface and hence available to avian predators.

The second benefit concerns smaller species of petrels and shearwaters (Procellariidae) which may not be able to target certain prey fish due to their relatively small body size and therefore opportunistically feed on floating scales and tissue [16,23,28]. Larger predators can injure more fish within a school than they capture per attack [29], leading to excised tissue being available for consumption by the birds. This hypothesis has been proposed to explain the association of Parkison's petrel (*Procellaria parkinsoni*) with melon-headed whales (*Peponocephala electra*) and false killer whales (*Pseudorca crassidens*), with the whales dismembering large prey at the surface and leaving food scraps in the process [16].

The third potential benefit that could facilitate these associations is related to a disruption of the collective defence of fish schools by the subsurface predators. Many defensive benefits for individuals in a group such as the confusion effect [30,31], rely on cohesive organization [32]. Predators interfere with these defensive mechanisms by reducing cohesion through successive attacks [33]. In the context of multi-species predator aggregations, Cape gannets (*Morus capensis*) were more than twice as successful at catching fish when attacks by other predators occurred in the 15 s prior to their attack [3]. An associated individual-based model showed that school cohesion (measured in terms of changes in the schools radius—the distance of each fish to the school's centroid over time) was reduced by successive predator attacks (attacks from either subsurface or avian predators [3,34]. Although this explanation is likely, direct measures of prey cohesion in relation to predator attacks are needed to demonstrate the causal effect of reduced cohesion. Otherwise, an alternative explanation could be that both subsurface and avian predators exploit favourable conditions (i.e. states of reduced prey cohesion) that are not caused by attacks, but rather, for example, via natural variation in prey speed [35]. Currently, there are no studies that directly quantified prey cohesion in the context of multi-species predator aggregations.

In this paper, we investigate the question of whether Indo-pacific sailfish (*Istiophorus platypterus*) create foraging opportunities for terns (*Onychoprion* sp*.*) when attacking schools of anchovies (*Encrasicholina* sp.) and sardinellas (*Amblygaster* sp*.*). We observed that sailfish attacks on fish schools frequently preceded attacks by terns which led to the hypothesis that sailfish created a by-product benefit for terns by reducing school cohesion and increasing prey vulnerability. First, we measured how often sailfish attacks directly preceded tern attacks, then we quantified the polygon outline of the prey school (as a proxy for cohesion) using high-resolution drone footage at regular time intervals before tern attacks. This was done to test whether bird attacks were preceded by sudden decreases in cohesion. Subsequently, we measured the polygon outline of the prey school before, during, and after a sailfish attack to test whether sailfish attacks resulted in sudden decreases in prey school cohesion. Finally, we directly related the polygon area of the prey school at the moment of the tern attack to the timing of the sailfish attacks, to test whether sailfish attacks were likely to create a by-product benefit for terns.

## 2. Methods

We collected high resolution drone video footage (DJI P4P pro v. 2.0, 2.7 k, 60 fps, 5–30 m) of sailfish and terns hunting schools of small prey fish, most likely anchovies (*Encrasicholina* sp.) and sardinellas (*Amblygaster* sp*.*) [36] in 2023 (21–27 August), off the eastern coast of the Malaysian peninsula in the South China Sea (2°56'15.4″ N 103°42'14.1″ E) (electronic supplementary material, video S1). Both predator species can reliably be encountered during this time off the east coast of the Malaysian peninsula. Sailfish were located opportunistically, by approaching seabird aggregations that preyed on the same schooling fish. The only seabirds that were observed feeding were of the genus *Onychoprion*, either sooty (*O. fuscatus*) or bridled (*O. anaethetus*) terns. Other seabirds (i.e. lesser frigate birds, *Fregata ariel*) occasionally accompanied sailfish, although they were never observed feeding. Five different fish schools were filmed in total where sailfish and terns were both attacking the same prey school. From this footage, we selected approximately 18 min where conditions were optimal (i.e. little to no wave or wind action, and minimal glare) in order to accurately measure school cohesion (five videos between 92 and 327 s).

**Figure 1.**
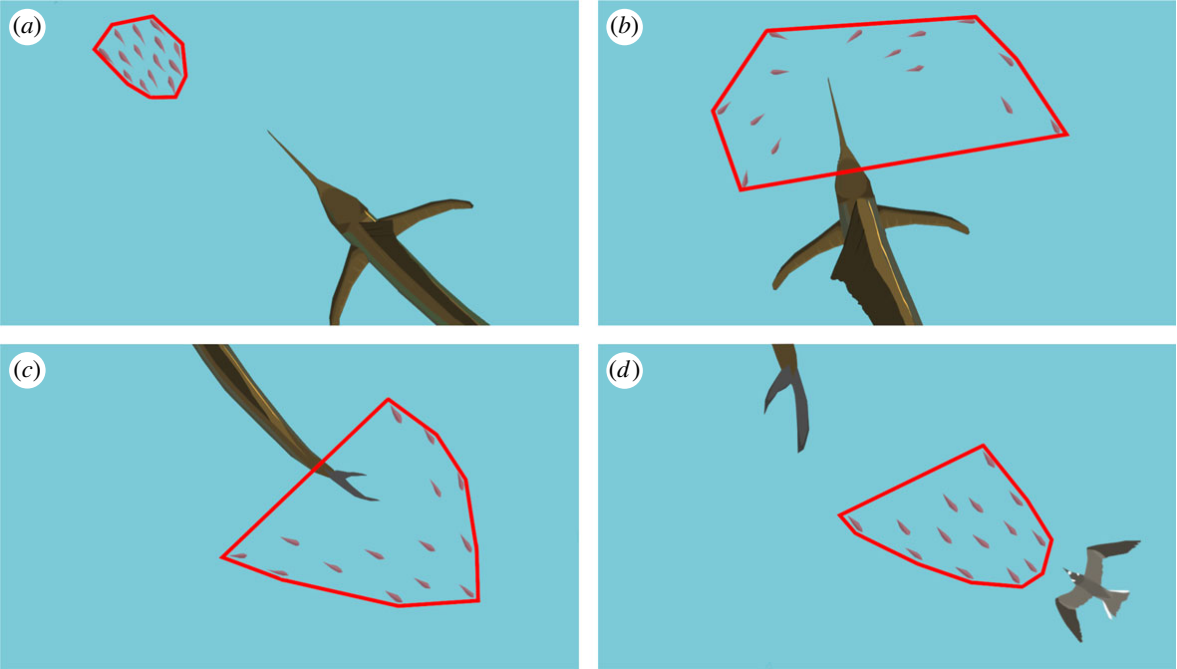
The *polygon size* (area of convex polygons) was used as a measure for school cohesion. Top: (*a*) point before a sailfish attack (t-1), (*b*) point of attack by a sailfish (t0). Bottom: (*c*) point before a bird attack (t-0.5), (*d*) point of a bird attack (t0). The initial stage (either t-3.5 or t-2) was set as 100% to make footage from different filming heights and angles comparable between samples.

Within the videos, we determined the time of every attack (made by sailfish (*N* = 126) or terns (*N* = 60)) that occurred. Sailfish attacks were identified by rapid slashes of the rostrum at prey schools [37]. Due to the small size of prey groups that were encountered, sailfish only attacked one at a time. Bird attacks were identified by swoops towards the ocean's surface and subsequent contact of their bill with the water. Capture success could only be assessed for seabird attacks, as captured prey was clearly visible in their beaks after an attack. To test our question related to the interaction between sailfish and seabirds we analysed the 32 attacks by terns which were preceded by sailfish attacks as well as the corresponding 32 sailfish attacks. These attacks were spread across the five fish schools, with up to 17 terns within a frame, however, although this is likely only a subset of total flock size in the area, we cannot exclude the possibility of pseudo-replication, as birds were not individually identifiable. To quantify prey behaviour, we exported still-frames out of the video, using VirtualDub2 (v. 1.10.5), and measured the area of the fish school using a convex polygon in ImageJ (*polygon size*) [38]. A convex polygon was used given the top-down, two-dimensional images of the drone. This measure was used as a proxy for school cohesion (figure 1; electronic supplementary material, image 1) [32]. This was done at the point of each bird attack, as well as each half second prior to this (up until 3.5 s before the attack). The same method was used to measure the impact of sailfish attacks on the prey school. Here we measured *polygon size* at the point of attack and 2 s prior and after the attack. Due to the small size of the prey fish (< 15 cm) as well as waves and light reflections, which occasionally restricted our view into the water, *polygon size* was likely underestimated, as some individual fish after sailfish slashes may have remained undetected.

As the height of the drone, distance to the fish school, as well as angle of the camera, varied between schools, we used the relative *polygon size*, enabling us to compare prey behaviour throughout a recording. The *polygon size* at the first measurement point of each series (either −3.5 or −2 s) was set as 100%, as the fish school was still unaffected by the approaching predators.

For all statistical analyses we used R (v. 4.2.2) [39]. To test at which time points *polygon size* significantly increased we used a Friedman test with Bonferroni post-hoc tests. We performed a linear regression to investigate the interaction between *polygon size* at the time point of tern attack and the time after a sailfish attack.

## 3. Results

**Figure 2.**
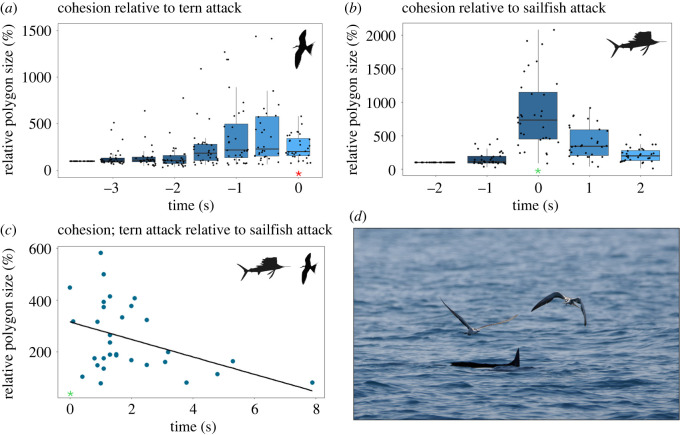
School cohesion is decreased by sailfish attacks, terns use these windows of dispersal to perform their attacks. (*a*) The relative size of the fish school polygon at the moment of a tern attack (t0/* in red) and during the 3.5 s before the attack; polygon size is relative to that at time −3.5 s which was set at 100%. Three outliers that exceeded 1500% were excluded from the figure to improve readability, but were included in the analysis. (*b*) Relative size of the fish school polygon in relation to sailfish attacks (t0/* in green); polygon size is relative to the initial size before the attack at time −2.0 s which was set at 100%. (*c*) Relative size of the fish school polygon at the time of a tern attack relative to when it occurred after a sailfish attack (t0/* in green), the trend line describes the linear regression. (*d*) Interaction between sailfish and terns as seen from the boat (image copyright Max Licht).

Out of 32 bird attacks that were directly preceded by sailfish attacks, 56% of these attacks occurred within 1.5 s after the sailfish attack. *Polygon size* increased significantly before a tern attack (Friedman *χ*² = 71.603, d.f. = 7, *p*-value = < 0.0001, figure 2*a*). Shortly before the tern attack (at times −1.5, −1, −0.5 s and during the attack at time 0 s) the *polygon size* was significantly larger than the school's initial *polygon size* (at time −3.5 s) (Bonferroni corrected *post-hoc* pairwise comparisons, all *p* < 0.001, figure 2*a*).

Sailfish attacks significantly increased *polygon size* (Friedman *χ*² = 81.61, d.f. = 4, *p*-value = < 0.0001, figure 2*b*). *Polygon size* at the time of a sailfish attack, and up to 2 s afterwards, were significantly larger than the initial size (at −2 s) (Bonferroni corrected post-hoc pairwise comparisons, all *p* ≤ 0.0001). In fact, *polygon size* at the moment of sailfish attack was on average six times larger than its initial size (figure 2*b*).

The sooner a tern attack took place after a sailfish attack, the larger the polygon size (figure 2*c*; *F*_1, 29_ = 6.015, *p* = 0.02044). One tern attack was excluded from the analysis as a sailfish attacked 0.2 s after the tern. Tern capture success was not analysed in relation to relative polygon size because there were only six successful attacks among a total of 32 attacks.

## 4. Discussion

### (a) By-product benefit

We found that attacks of sailfish reduced prey school cohesion, which in turn triggered tern attacks. The sooner terns attacked after a sailfish attack, the lower the school cohesion was. Despite the large number of publications on open ocean multi-species predator aggregations [5,6,12,16,40,41], evidence of mechanisms creating between-species benefits are rare. By quantifying the spatio-temporal properties of the prey school in relation to subsurface and aerial attacks we identified a mechanism which likely generated a benefit for terns, deepening our understanding of the formation and maintenance of multi-predator aggregations. Previous work on sailfish has identified mechanisms by which attacks from predators of the same species can provide benefits to each other, such as injuring more prey than are captured per attack [29]. These mechanisms can be classified as by-product mutualisms, as individuals generate and benefit from the same mechanism [42]. Here, sailfish generated a foraging opportunity for terns by reducing prey cohesion, and because the interaction causes no discernible benefit to the sailfish, it is probable that it represents a commensal or kleptoparasitic relationship, rather than a mutualistic one.

The relationship between predators in multi-species predator aggregations in the open ocean has been notoriously difficult to investigate, due to the challenges of closely monitoring attacks and captures in these interactions [43]. Classification of species as ‘producers’ or ‘scroungers’ has largely relied on the arrival time of the interaction partners and anecdotal observations from above and below the surface [12,40,41]. Two studies that quantified detailed data on attack and capture rates of one or two predator species, proposed a mutualistic and a kleptoparasitic relationship, respectively [3,4]

### (b) School cohesion affects predators and vice versa

Most studies on the between-predator interactions of seabirds with subsurface predators ignore the possibility that different predator species are attracted to the same prey independently, rather than one species providing an opportunity for another. Here, we show that tern attacks are usually preceded by a decrease in school cohesion. Attacking during low cohesion presumably results in greater capture success, and thus could maximize energy efficiency. However, we did not observe enough successful fish captures to address the question of whether greater polygon size and thus lower cohesion, results in greater capture success. A reduction in school cohesion by predators has previously been reported for menhaden (*Brevoortia tyrannus*), where the predators formed a line during attacks delaying the reformation of the school after an attack [44]. The decrease in cohesion from an attack, coupled with the attraction of predators to attack schools with low cohesion, could trigger a succession of predator attacks [34]. Our results provide direct evidence of this in the context of multi-species predator aggregations, as initially proposed by Thiebault *et al*. and Lett *et al*. [3,34]. Further, short time intervals with rapidly increased attack rates on prey schools, followed by periods of less activity have previously been reported in multiple systems [3,23].

### (c) Polygon increases not caused by sailfish

The initial polygon size of the prey school did not change significantly until 1.5 s before a bird attack and only 56% of tern attacks were preceded by sailfish attack within 1.5 s, leaving a share of decreased prey cohesion unexplained. Candidate factors that could cause prey cohesion to decrease apart from the influence of sailfish attacks include conflicts about movement directions within the fish school caused by sailfish nearby [45] and looming stimuli from the approaching bird [46].

We used convex polygons as a simple measure of school cohesion, which itself is a proxy for the occurrence of isolated individual fish given that most predators of fish schools target isolates [47–49] Ideally, conditional on perfect recording conditions for the prey school and its individual members, individual isolation should be measured directly by quantifying the location of each member of the prey school and using the nearest neighbour distance or inter-individual distance as a measure of individual isolation. We were unable to use this method as sun glares occasionally obstructed the view of individual fish, as well as the small size of the prey species in this study system.

### (d) Interdependence

Seabird species like sooty terns and frigate birds, that do not perform plunge attacks, may strongly rely on subsurface predators for prey capture [18,25]. In one observation, we witnessed a sailfish split a small school away from a large school of over 10 000 individual prey fish. Interestingly, the terns followed the smaller school of prey with the sailfish (despite the potential competition) and no individuals stayed with the large school which was still at the surface and seemingly available to the birds. This suggests that the seabirds might have learned to associate the presence of sailfish with favourable foraging conditions [50,51]. Although we do not know the full extent to which seabirds rely on subsurface predators for all foraging efforts, the presence of the latter could make foraging more effective, as has been suggested for other seabird species [19]. In this context it would be interesting to compare feeding rates of seabirds in the presence and absence of underwater predators on the same prey species. This could be especially relevant during the breeding season because of increased energy demands [41].

## 5. Conclusion

Building on the pioneering work of Thiebault *et al*. [3] we provide direct evidence for the effects of predator attacks on the cohesion of prey groups and how this in turn could facilitate the association of avian predators with large teleost predators in the open ocean. Reports of associations of seabirds with large teleost predators and dolphins are very common [3,5,13–19] and provide many opportunities for further studies of the mechanisms underlying the generation of foraging benefits between species.

## Data Availability

The data are provided in the electronic supplementary material [52].
